# The Fear Avoidance Model predicts short-term pain and disability following lumbar disc surgery

**DOI:** 10.1371/journal.pone.0193566

**Published:** 2018-03-05

**Authors:** Faris A. Alodaibi, Julie M. Fritz, Anne Thackeray, Shane L. Koppenhaver, Jeffrey J. Hebert

**Affiliations:** 1 Department of Rehabilitation Science, College of Applied Medical Sciences, King Saud University, Riyadh, Saudi Arabia; 2 School of Health, Department of Physical Therapy, University of Utah, Salt Lake City, UT, United States of America; 3 Baylor University Doctoral Physical Therapy Program, Waco, TX, United States of America; 4 School of Psychology and Exercise Science, Murdoch University, Murdoch, Western Australia, Australia; 5 Faculty of Kinesiology, University of New Brunswick, New Brunswick, Canada; Brown University, UNITED STATES

## Abstract

**Objective:**

To examine the prognostic value of the Fear Avoidance Model (FAM) variables when predicting pain intensity and disability 10-weeks postoperative following lumbar disc surgery.

**Methods:**

We recruited patients scheduled for first-time, single level lumbar disc surgery. The following aspects of the FAM were assessed at preoperative baseline and after 10 postoperative weeks: numeric pain rating scale (0–10) for leg and back pain intensity separately, Pain Catastrophizing Scale (PCS), Fear Avoidance Beliefs Questionnaire (FABQ), Beck Depression Inventory (BDI), Oswestry Disability Questionnaire (ODI), and the International Physical Activity Questionnaire (IPAQ). Multivariate regression models were used to examine the best combination of baseline FAM variables to predict the 10-week leg pain, back pain, and disability. All multivariate models were adjusted for age and sex.

**Results:**

60 patients (30 females, mean [SD] age = 40.4 [9.5]) were enrolled. All FAM measures correlated with disability at baseline. Adding FAM variables to each of the stepwise multiple linear regression model explained a significant amount of the variance in disability (*Adj*. *R*^*2*^ = .38, *p* < .001), leg pain intensity (*Adj*. *R*^*2*^ = .25, *p* = .001), and back pain intensity *Adj*. *R*^*2*^ = .32, *p* < .001 at 10-weeks). After adjusting for age and gender, BDI and FABQ-work subscale were the only significant predictors added to each of the prediction models for the 10-week clinical outcome (leg pain, back pain, and ODI).

**Conclusion:**

BDI and FABQ-work subscale variables are associated with baseline pain intensity and disability and predict short-term pain and disability following lumbar disc surgery. Measuring these variables in patients being considered for lumbar disc surgery may improve patient outcome.

## Introduction

Sciatica is a common disorder characterized by pain radiating to the leg, most commonly caused by lumbar disc herniation (LDH).[1, 2] Although the natural history of sciatica and LDH is favorable, many patients undergo lumbar disc surgery if conservative treatment fail and there are persistent symptoms with imaging confirmed disc herniation.[3] The outcomes of surgery depend on the appropriate and careful selection of patients.[4]

Although surgical patient selection has most commonly focused on physical examination findings, there appears to be a growing appreciation of psychosocial factors in patients undergoing operative and non-operative treatment for LDH.[5–10] The fear avoidance model (FAM) is a psychosocial model that seeks to understand the role of cognitive, behavioral, physical, and emotional factors in persistence of pain and disability.[11, 12] However, research examining the prognostic value of the FAM has predominantly focused on non-operative populations (i.e., patients with non-specific low back pain).[12] Preliminary evidence indicates that the FAM may be relevant to specific LBP conditions including the prediction of surgical outcomes.[10, 12–15]

FAM is one of the most widely reported models used to explain the development of chronic musculoskeletal pain and disability. [11, 12] FAM theorizes experiencing pain after an insult can be reinforced by a negative thinking (catastrophizing) and avoidance of physical activity due to fear of pain. [12] With time and maladaptive behaviors, physical activities decline, disability increases, and depression may develop.[12] As these conditions compound, pain and disability appear to increase. Previous studies of lumbar disc surgery have investigated the prognostic value of some aspects of the FAM such as pre-operative pain, pain-related fear, avoidance and maladaptive behaviors, anxiety, depression, and functional disability. Den Boer et al. followed 277 patients undergoing LDH surgery for 6 months. [16] They found that fear of movement/reinjury predicted more disability and more severe pain at 6 weeks and more severe pain at 6 months postoperatively. Likewise, passive coping strategies predicted more disability at 6 months postoperatively. Generally, negative associations between these psychosocial variables and clinical outcomes have been reported. [9, 15, 16] Examining FAM variables among patients undergoing lumbar disc surgery may assist in developing intervention strategies. Pain catastrophizing, fear-avoidance beliefs and physical activity level are FAM factors associated with chronic non-specific LBP, but their role in patients undergoing LDH surgery is less well-understood.[15, 17–22]

Therefore, the purpose of this study was to explore the cross-sectional and longitudinal associations of pre-operative FAM measures (pain catastrophizing, fear-avoidance, depression and physical activity) with leg pain, back pain, and pain related disability both before and after surgery among patients with LDH confirmed by imaging scheduled to undergo first time single-level discectomy or microdiscectomy. We hypothesized that the preoperative FAM measures would be positively associated with preoperative pain and disability and predict clinical outcomes after 10 postoperative weeks.

## Methods

### Participants and study design

This was a planned secondary analysis of a randomized control trial comparing two rehabilitation programs following disc surgery (received ethical approval from the institutional review board of the University of Utah, trial registration ID: NCT00894972).[23] The original study did not find between-group differences in the clinical outcomes; therefore, all of the participants were combined into a single cohort for analysis in the current study. Study participants were patients between 18 and 60 years with imaging confirmed LDH and scheduled to undergo first time single-level discectomy or microdiscectomy. Potential participants were excluded if they had significant medical co-morbidities or conditions that prevented them from participation in a postoperative exercise program.

Recruitment of the participants took place between April 2009 and July 2012 from orthopedic and neurologic surgeon offices located in Salt Lake City, Utah. Baseline demographic, psychosocial and clinical measures were obtained less than 2 weeks before surgery. This information included age, gender, and body mass index (BMI) as well as several self-report measures related to the FAM. Details about examined the FAM and clinical measures in the study are provided below. Measures of clinical outcome were obtained after 10 postoperative weeks.

### FAM and clinical outcome measures

The FAM as described by Vlaeyen and Linton includes pain, pain catastrophizing, fear-avoidance, depression, physical activity, and disability as components of the model explaining chronic symptoms. [11, 12]

Leg pain and back pain intensity were assessed separately using Numeric Pain Rating Scales (NPRS). Patients rated their leg and back pain on an 11-point scale from zero (no pain) to 10 (worst imaginable pain). Pain measures were taken for current, best, and worst pain in the past 24 hours. The three scores were then averaged to represent each patient’s average pain. NPRS has been shown to be reliable, valid, and responsive for individuals with LBP and for postoperative population.[24,25]

Pain catastrophizing was measured using the 13-item pain catastrophizing scale (PCS).[26] Each item asks about the frequency of negative thoughts and feeling while experiencing pain (e.g., “It’s terrible and I think it’s never going to get any better”) and is rated from zero (not at all) to 4 (all the time). This scale captures three different components of pain catastrophizing (helplessness, rumination, and magnification). The PCS is a valid and reliable measure for both genders and different ages.[27–29] Greater pain catastrophizing is associated with persistent postoperative pain.[30–32] Sullivan and colleagues identified a PCS score of >30 as representing clinically-relevant pain catastrophizing.[26]

Fear-avoidance beliefs were measured using the fear avoidance beliefs questionnaire (FABQ).[20] The FABQ comprises two subscales; one measures the potential influence of fear-avoidance beliefs on general physical activity (FABQ-PA) and the other on work-related activity (FABQ-W). Cut-off scores of >13 for the FABQ-PA and score of >29 for the FABQ-W were predictive of poor outcome.[33, 34] The FABQ is a reliable measure that has been shown to be predictive of the transition from acute to chronic LBP.[22, 35]

Depression was self-reported with the beck depression inventory II (BDI).[36] The BDI includes 21 questions that measure depression symptoms. Each answer is scored from 0 to 3 giving a total possible score of 63. According to the BDI-II manual, a total score of 0 to 13 represents no depression; 14 to 19, mild depression; 20 to 28, moderate depression; and, more than 28 represents severe depression. The BDI has good psychometric properties and has been shown to have good predictive validity in patients with chronic pain. [37–38]

Physical activity (PA) level was measured using the short form of the international physical activity questionnaire (IPAQ).[39] IPAQ is a self-report questionnaire that asks questions about walking as well as moderate and vigorous physical activities in the last 7 days. Individuals are classified into low, moderate, or high activity levels. The IPAQ has acceptable validity and reliability to measure physical activity.[37] In this study, we dichotomized PA level into active (high or moderate activity level group) or inactive (low activity level group).

Low back pain-related disability was measured with the modified oswestry disability questionnaire (ODI).[40,41] The ODI comprises 10 questions that rate the impact of back pain on daily functional activities. Each answer is scored from 0 to 5, and total possible score is summated to a total possible score of 100. Higher scores indicate greater disability. The ODI has been shown to be valid, reliable and has a high level of responsiveness.[41,42]

At the 10^th^ postoperative week follow-up, leg pain, back pain, and disability were assessed with the same baseline scales and measures (i.e., NPRS and ODI).

### Statistical analysis

Data analysis was performed using the statistical package for the social sciences (SPSS 22.0, Chicago, IL). To summarize and describe the sample at baseline and 10-weeks, descriptive statistics including mean ($\bar{x}$) with standard deviation (SD), median (*M*) with interquartile ranges (IQR), and counts with percentages were performed.

To examine the bivariate relationships between baseline FAM variables and baseline leg pain, back pain and disability, the Pearson product moment correlation coefficient (*r*) was used. Coefficient (*r*) between -1 to -.5 or .5 to 1 were considered strong correlation, -.5 to -.3 or .3 to .5 were considered medium correlation, and from -.3 to -.1 or .1 to .3 were considered weak correlation.

Stepwise multiple regression analyses were conducted to examine the best combination of FAM variables to predict the 10-week postoperative outcomes (leg pain [NPRS], back pain [NPRS], and disability [ODI]) after controlling for age and gender. In step one age and gender were force entered and in step two the FAM variables were entered using stepwise method (Criteria: Probability-of- F -to-enter < = .05, Probability-of- F -to-remove > = .10). Adjusted *R*^*2*^ (Coefficient of determination) was calculated for each of the models to examine how much variance in the dependent variable was explained by the independent variables that were entered to each of the regression models.

All analyses were based on two-tailed *p*-value tests and the significance level was set at (0.05). Listwise (in the regression analyses) and pairwise deletion (in bivariate correlation analysis) were used in the case of missing values.

## Results

### Patients’ baseline characteristics

Baseline measures were available for 60 patients (30 males, 50%). Patients’ age ranged from 21 to 56 years old ($\bar{x}$ = 40.4, *SD* = 9.5), BMI from 19.6 to 48.7 ($\bar{x}$ = 29.5, *SD* = 7.0). The median of the symptoms’ duration before surgery was 141 days ($\bar{x}$ = 193.7, *SD* = 190.7). 11 patients were out of work and 20 patients were working with restrictions and reduced hours due to current back problems (average days of missed work were 11 days, *SD* = 20.4). Fifty patients had symptoms distal to the knee. Thirty-seven patients complain of numbness symptoms half of the time or more. Descriptive statistics of other baseline and the 10-week measures are displayed in Table 1.

**Table 1:** Descriptive statistics of baseline and the 10 week measures.

Measure		$\bar{x}$ (SD)
Baseline leg pain	Female	5.8 (2.3)
	Male	5.3 (2.7)
Baseline back pain	Female	4.6 (2.4)
	Male	3.9 (2.7)
Baseline ODI	Female	48.1 (14.7)
	Male	38.9 (14.7)
Baseline FABQ-PA	Female	17.1 (5.6)
	Male	18.0 (6.7)
Baseline FABQ-W	Female	16.9 (11.4)
	Male	18.1 (12.1)
Baseline PCS	Female	19.6 (11.3)
	Male	18.9 (10.9)
Baseline BDI	Female	12.5 (9.2)
	Male	8.4 (6.0)
10 week leg pain	Female	1.67 (1.8)
	Male	0.8 (1.34)
10 week back pain	Female	2.2 (2.0)
	Male	1.2 (1.6)
10 week ODI	Female	18.8 (17.8)
	Male	10.0 (13.6)

PCS: Pain Catastrophizing Scale, FABQ-PA: the Fear Avoidance Belief Questionnaire (physical activity subscale), FABQ-W: the Fear Avoidance Belief Questionnaire (work subscale), IPAQ: the International Physical Activity Questionnaire, BDI: Beck Depression Inventory, ODI: Oswestry Disability Questionnaire

Fourteen patients (23%) had clinically relevant levels of catastrophizing (PCS>30) [26]. Forty-four (73%) of the patients had no depression symptoms, 9 (15%) had mild depression, 4 (6.7%) had moderate depression, and 3 (5%) had severe depression (BDI>28). Ten (16.7%) patients scored above 29 on the FABQ-work subscale (previously reported cut-off [33,34]) and 42 (70%) patients scored above 14 on the FABQ-physical activity subscale (cut-off score [43,44]). Baseline IPAQ classified patients as low activity for 34 (57%) patients and medium or high activity for 23 (38%) patients. Three patients had missing IPAQ scores.

### Clinical outcome measures

The 10-week clinical outcomes were available for 55 patients. Their average 10-week ODI was 14.3 (*SD* = 16.3), leg pain was 1.2 (*SD* = 1.6), and back pain was 1.7 (*SD* = 1.9).

### Bivariate associations between demographics, baseline FAM measures, and the baseline pain and disability

All of FAM measures correlated with preoperative disability (ODI). Pain catastrophizing (PCS) correlated with preoperative leg pain, back pain, and disability. Strongest correlations were seen between back pain and PCS (*r* = .49, *p* < .001), and between back pain and BDI (*r* = .46, *p* < .001). Younger age associated significantly with higher baseline back pain (*r* = -.43, *p* = .001) and with higher baseline ODI (*r* = -.36, *p* < .01). Likewise, female gender was associated with higher baseline ODI (*r* = -.30, *p* = .02) and higher baseline BDI (*r* = -.26, *p* = .05). Baseline BMI did not show any significant correlation with the baseline FAM variables. Therefore, all subsequent regression analyses were adjusted for age and gender.

### Predictors of the 10-week postoperative leg pain, back pain, and disability

We ran three stepwise multiple linear regression analyses to determine the unique variance of the 10-week clinical outcome (leg pain, back pain, and ODI) accounted for by the best significant combination of FAM variables. Each model was adjusted for age and gender (force entered).

Final model explained a significant amount of the 10-week ODI variance, *Adj*. *R*^*2*^ = 0.38, *p* < .001. Among all of the FAM variables entered, only BDI (*b* = 1.02, *p* < .001) and FABQ-W (*b* = .34, *p* = .037) reached significance for predicting the 10-week ODI, Table 2 shows the predictors’ standardized coefficients (β) and the change in *R*^*2*^, from model 1 (adjusted variables, age and gender) to model 2 (addition of FAM variables).

**Table 2:** Stepwise multiple linear regression to predict the 10-week ODI.

Model	Predictors	*b*	*p*-value	*Adj*. *R*^*2*^
**1**	Age	-.22	.356	.03
	Gender	-5.78	.199	
**Final**	Age	-.07	.727	.38**
	Gender	-3.04	.412	
	BDI	1.02**	.000	
	FABQ-W	.34*	.037	

Dependent variable: 10-week postoperative ODI

*p < .05

**p < .01

PCS: Pain Catastrophizing Scale, FABQ-PA: the Fear Avoidance Belief

Questionnaire (physical activity subscale), FABQ-W: the Fear Avoidance Belief

Questionnaire (work subscale), IPAQ: the International Physical Activity

Questionnaire, BDI: Beck Depression Inventory, ODI: Oswestry Disability Questionnaire

Likewise, a significant amount of the 10-week leg pain (NPRS) variance was explained in the final model, *Adj*. *R*^*2*^ = 0.25, *p* = .001. In the final model, none of the FAM variables reached significance to predict the 10-week leg pain except BDI (*b* = .07, *p* = .015) and FABQ-W (*b* = .04, *p* = .032). Table 3 shows the results of the stepwise multiple linear regression analysis to predict the 10-leg pain.

**Table 3:** Stepwise multiple linear regression to predict the 10-week leg pain.

Model	Predictors	*b*	*p*-value	*Adj*. *R*^*2*^
**1**	Age	-.02	.322	.03
	Gender	-.56	.204	
**Final**	Age	-.01	.633	.25**
	Gender	-.41	.311	
	BDI	.07*	.015	
	FABQ-W	.04*	.032	

Dependent variable: 10-week postoperative leg pain

*p < .05

**p < .01

PCS: Pain Catastrophizing Scale, FABQ-PA: the Fear Avoidance Belief

Questionnaire (physical activity subscale), FABQ-W: the Fear Avoidance Belief

Questionnaire (work subscale), IPAQ: the International Physical Activity

Questionnaire, BDI: Beck Depression Inventory, ODI: Oswestry Disability Questionnaire

The third stepwise multiple linear regression analysis was performed to predict the 10-week back pain (NPRS). Age was significant when entered with gender in the first step (*b* = -.05, *p* = .04); however, it lost its significance in the second step. Entering FAM variables in the second step explained a significant amount of the 10-week postoperative back pain variance, *Adj*. *R*^*2*^ = 0.32, *p* < .001. However, none of the FAM predictors remained in the final model except BDI (*b* = .08, *p* = .008) and FABQ-W (*b* = .04, *p* = .021). Table 4 shows the results of the hierarchical regression analysis to predict the 10-back pain.

**Table 4:** Stepwise multiple linear regression to predict the 10-week back pain.

Model	Predictors	*b*	*p*-value	*Adj*. *R*^*2*^
**1**	Age	-.05*	**.044**	.08*
	Gender	-.45	.351	
**Final**	Age	-.04	.110	.32**
	Gender	-.27	.532	
	BDI	.08**	.008	
	FABQ-W	.04*	.021	

Dependent variable: 10-week postoperative back pain

*p < .05

**p < .01

PCS: Pain Catastrophizing Scale, FABQ-PA: the Fear Avoidance Belief

Questionnaire (physical activity subscale), FABQ-W: the Fear Avoidance Belief

Questionnaire (work subscale), IPAQ: the International Physical Activity

Questionnaire, BDI: Beck Depression Inventory, ODI: Oswestry Disability Questionnaire

## Discussion

Our results supported the relationship between preoperative BDI and FABQ-W variables and the 10-week clinical outcome following surgery for LDH. Moderate relationships were seen between many of the FAM measures and preoperative pain and disability. Baseline disability and pain catastrophizing correlated significantly with all of the included FAM measures. Being young associated with more disability at baseline and more back pain both at baseline and at week 10 following surgery. Being female was associated with more disability and depression at baseline and worse clinical outcomes following surgery. This finding was supported by previous research findings that being female is associated with poorer LDH surgical outcomes.[16, 45, 46] Therefore, we adjusted all the subsequent regression analyses for age and gender.

Baseline depression and work-related fear-avoidance beliefs were the only FAM variables that explained significant amounts of the three 10-week outcome scales and measures (leg pain, back pain, and disability). Moderate to strong correlations between baseline depression, pain catastrophizing, and back pain might prevent the last two from reaching statistical significance to predict the postoperative outcomes. Previous studies supported the impact of both fear and depression on LDH surgical outcomes.[9, 16, 46–49] Our results also were similar to previous studies in LDH patients showing higher baseline back pain increased the risk of unfavorable surgical outcomes.[9, 50]

A number of limitations need to be taken into account when interpreting these results. LDH is defined as a displacement of the disc materials, however, there are many sub-categorizations under this diagnosis that we did not try to control for or influence the surgeon’s decision for surgery. Also, the relatively small sample size included in this analysis may question the accuracy of these results. In addition, our analysis included only one follow-up time point and was relatively short (i.e., 10 weeks postoperative). Therefore, we were unable to compare our results with another and longer follow-up. Lastly, all the measures in this study were self-report measures. Although self-report measures are easier to use, response biases may distort the results. Therefore, future prospective studies with larger sample sizes, longer and multiple follow-up time points are warranted.

To our knowledge, this is the first study to use FABQ and PCS measures to predict LDH surgical outcomes. Our results supported the association between baseline FAM measures and preoperative functional disability. Our results also supported the impactfullness of both depression and work-related fear-avoidance beliefs on LDH postsurgical leg pain, back pain, and functional disability.

## Conclusion

FAM measures have moderate relationships with preoperative pain and disability. Preoperative depression and work-related fear-avoidance beliefs were able to significantly explain the 10-week clinical outcome variances (leg pain, back pain, and disability). In general, higher baseline back pain was associated with unfavorable surgical outcomes. These data may enhance patient selection and counseling patients considered for lumbar disc surgery regarding expected clinical outcome.

## Supporting information

S1 Dataset FAM_minimal data set. (XLSX)
